# Expanding conceptualizations of harm reduction: results from a qualitative community-based participatory research study with people who inject drugs

**DOI:** 10.1186/s12954-017-0145-2

**Published:** 2017-05-12

**Authors:** L. M. Boucher, Z. Marshall, A. Martin, K. Larose-Hébert, J. V. Flynn, C. Lalonde, D. Pineau, J. Bigelow, T. Rose, R. Chase, R. Boyd, M. Tyndall, C. Kendall

**Affiliations:** 1 Élisabeth Bruyère Research Institute, 43 Bruyère Street, Annex E, Ottawa, Ontario K1N 5C8 Canada; 2 Social Development Studies & School of Social Work, Renison University College, University of Waterloo, 240 Westmount Road North, Waterloo, Ontario N2L 3G4 Canada; 3 Ottawa Hospital Research Institute, 1053 Carling Avenue, Ottawa, Ontario K1Y 4E9 Canada; 4 School of Social Work, Faculty of Social Sciences, Laval University, Charles de Koninck Hall, 1030, avenue des Sciences-Humaines, Quebec, G1V 0A6 Canada; 5 Department of Social Work, University of Gothenburg, Sprängkullsgatan 23, 405 30 Gothenburg, Sweden; 6 PROUD Community Advisory Committee, Ottawa, Ontario Canada; 7 Department of Community Health Sciences, Faculty of Health Sciences, University of Manitoba, 750 Bannatyne Ave, Winnipeg, Manitoba R3E 0W2 Canada; 8 Sandy Hill Community Health Centre, 221 Nelson Street, Ottawa, Ontario K1N 1C7 Canada; 9 BC Centre for Disease Control, 655 W 12th Avenue, Vancouver, British Columbia V5Z 4R4 Canada; 10 Department of Family Medicine, Faculty of Medicine, University of Ottawa, 43 Bruyère Street, (375) Floor 3JB, Ottawa, Ontario K1N 5C8 Canada

**Keywords:** Harm reduction, Community-based participatory research, People with lived experiences, Injection drug use, Agency, Self-care, Community engagement, Health and social services, Equity

## Abstract

**Background:**

The perspectives of people who use drugs are critical in understanding why people choose to reduce harm in relation to drug use, what practices are considered or preferred in conceptualizations of harm reduction, and which environmental factors interfere with or support the use of harm reduction strategies. This study explores how people who inject drugs (PWID) think about harm reduction and considers the critical imperative of equity in health and social services delivery for this community.

**Methods:**

This community-based participatory research study was conducted in a Canadian urban centre. Using a peer-based recruitment and interviewing strategy, semi-structured qualitative interviews were conducted by and with PWID. The Vidaview Life Story Board, an innovative tool where interviewers and participant co-construct a visual “life-scape” using a board, markers, and customized picture magnets, was used to facilitate the interviews. The topics explored included injection drug use and harm reduction histories, facilitators and barriers to using harm reduction strategies, and suggestions for improving services and supports.

**Results:**

Twenty-three interviews with PWID (14 men and 9 women) were analysed, with a median age of 50. Results highlighted an expanded conceptualization of harm reduction from the perspectives of PWID, including motivations for adopting harm reduction strategies and a description of harm reduction practices that went beyond conventional health-focused concerns. The most common personal practices that PWID used included working toward moderation, employing various cognitive strategies, and engaging in community activities. The importance of social or peer support and improving self-efficacy was also evident. Further, there was a call for less rigid eligibility criteria and procedures in health and social services, and the need to more adequately address the stigmatization of drug users.

**Conclusions:**

These findings demonstrated that PWID incorporate many personal harm reduction practices in their daily lives to improve their well-being, and these practices highlight the importance of agency, self-care, and community building. Health and social services are needed to better support these practices because the many socio-structural barriers this community faces often interfere with harm reduction efforts. Finally, “one size does not fit all” when it comes to harm reduction, and more personalized or de-medicalized conceptualizations are recommended.

## Background

Harm reduction among people who use drugs originally grew from informal grassroots practices, beginning with the illegal distribution of sterile syringes by activists and front-line workers [1–7]. These practices were increasingly taken up by public health stakeholders as an alternative to abstinence-focused practices because they were successful in reducing human immunodeficiency virus (HIV) transmission through injection drug use during the early stages of the epidemic [1, 6–9]. Through the 1980s and 1990s, harm reduction became institutionalized in many settings, yet in countries with more repressive laws it remained “a politicized form of action, reliant on civil disobedience” [1, 5, 8]. This is evident in comparing the situation between Canada and the USA. While public health institutions in Canada have widely incorporated harm reduction, repressive judicial control in the USA has led to the development of more pronounced informal practices and a greater politicization of harm reduction among communities of people who use drugs [1, 8].

While promoting harm reduction as necessary for the greater health of the population has allowed for pragmatic strides and expansion in the movement, it has also removed the control of harm reduction services from the communities who use and experience them [1, 8]. The adoption (some would even say co-option) of harm reduction through public health programs and policies has thus been problematic for communities of people who use drugs, as it has de-politicized harm reduction and reduced community agency and solidarity [1]. Whereas population-level goals of harm reduction have included reducing transmission of infectious diseases, preventing overdoses, decreasing other injuries related to drug use, or reducing crime, the importance of harm reduction to people who use drugs may extend beyond these goals to include a greater emphasis on agency and community building [10, 11].

As an “emerging public health perspective”, a multitude of definitions of harm reduction circulate in the literature [12, 13], and the use of harm reduction practices will vary according to the context of perceived harms. The International Harm Reduction Association [14] defines harm reduction as “policies, programmes and practices that aim to reduce the harms associated with the use of psychoactive drugs in people unable or unwilling to stop. The defining features are the focus on the prevention of harm, rather than on the prevention of drug use itself, and the focus on people who continue to use drugs”. Harm reduction has more recently evolved to include broader concerns such as the needs, preferences and values of people who use drugs. The United Nations Office on Drugs and Crime [15] has outlined harm reduction as important for reducing the adverse health and social consequences of drug use. Practices commonly referred to as harm reduction include education, opioid replacement therapy (e.g. methadone maintenance programs, also referred to as opioid substitution treatment), needle and syringe distribution, emphasizing routes other than injection for drug administration, counselling, naloxone distribution, sexually transmitted infection services, testing, wound care, vaccinations, social assistance, reducing injury or violence, and peer support, among others [15]. Note that some stakeholders may not consider these practices to be harm reduction, but rather as treatment interventions. Other definitions of harm reduction stem from outside public health conceptions, including harm reduction as a philosophy, a way of life, or a set of practices [16–18], all of which involve more of an emphasis on community. For instance, Gowan, Whetstone, and Andic [5] highlighted the importance of community building as a main purpose of the heroin users’ group described in their ethnographic study. Because stigmatization of the illicit drug user as “powerless” to a drug was considered an obstacle to employing harm reduction strategies in one’s life, one of the central practices of this group was to promote agency among its members in order to facilitate harm reduction actions.

In North America, current public health understandings of harm reduction tend to be narrow, medicalized, and situated within health and social service provision, which impedes broader thinking about non-medical aspects of well-being [5]. As current conceptualizations of harm reduction focus on reducing transmission of diseases, harm reduction services tend to focus on public safety rather than on prioritizing the broader or preferred needs of people accessing the services. People who use drugs may prefer that other harms they experience be addressed before the public health focus of reducing morbidity or mortality among service recipients. For instance, Harris and Rhodes [19] demonstrated that people who inject drugs (PWID) were more interested in acute health care advice such as venous access rather than the hepatitis C prevention information provided by practitioners. Beyond immediate needs of the individual, people who use drugs have also shown community-level thinking in how they conceive and practice harm reduction, espousing values of solidarity through altruistic beliefs and actions [10]. Furthermore, public health conceptions of harm reduction do not include the range of strategies that people who use drugs use to reduce harm in their daily lives. People with lived experiences are often left out of the conceptualization of harm reduction interventions, despite calls to include their voice in recommendations for harm reduction service delivery and implementation [15]. As such, we need to attend to who has the power to define harm reduction and how harm reduction services are structured and delivered, so as to ensure affected communities are meaningfully engaged in these processes. Although we acknowledge there are financial constraints with respect to harm reduction as public policy that may contribute to the differences between harm reduction as a concept and harm reduction programs and services, our study is focused on the conceptualization of harm reduction at the individual and collective community practice level as it relates to policy and programs.

### Harm reduction and self-care among people who use drugs

Traditionally, people who use drugs have been considered to be either morally flawed “deviants” or who lack free will due to the pathology of addiction [5, 20]. Such views have fostered a public perception that neglects the capacity for self-care by people who use drugs. Self-care refers to “a range of care activities deliberately engaged throughout life to promote physical, mental and emotional health, maintain life and prevent disease. Self-care is performed by the individual on their own behalf, for their families, or communities, and includes care by others. In the event of injury, disability or disease, the individual continues to engage in self-care, either on their own or in collaboration with healthcare professionals…” [21]. Although this definition is comprehensive, it is important to note that many multifaceted definitions of self-care exist and have evolved over time [21]. In addition, to understand the ways people who use drugs practice self-care, the role of agency is critical. Here agency is conceptualized as situated within social landscapes, “in which past routines are contextualized and future possibilities envisaged within the contingencies of the present moment” [22, 23]. Although there have been historical attempts to “strip” people who use drugs of agency, these individuals indeed apply self-care strategies within their daily conditions. A qualitative study on methadone diversion practices showed that people who use drugs prefer to have opportunities to self-regulate their harm reduction practices, despite being given very little flexibility to do so [11]. The fact that people who use drugs use self-care in spite of the many obstacles they face portrays a resilience that is often not credited to this community. Validation from support services has been shown to help people who use drugs recognize that they possess such strengths. For instance, Gowan et al. [5] found that receiving positive feedback through a heroin users’ support group reinforced members’ identities as self-caring individuals.

Although several qualitative studies have argued that self-care is common among people who use drugs, there has been minimal literature detailing the personal, day-to-day harm reduction practices that people who use drugs employ to mitigate harms arising from drug use [5, 24, 25]. People who use drugs also face a multitude of barriers (e.g. unstable housing, financial instability, physical and mental illnesses, criminalization), which interfere with exercising such self-care strategies. Hence, support for self-care practices may need to be better integrated into health and social services [5]. Moreover, the pervasive anti-drug discrimination and criminalization in society that contributes to drug users’ distrust of service providers and other authority figures [17, 24, 26, 27] needs to be considered. For instance, one study [24] identified that some people who use drugs employ self-care practices “at home” to treat medical injuries, such as abscesses or fractures, in order to avoid the need to seek out professional health care. Because of the ways drug users have been treated by people in positions of authority, harm reduction should be conceptualized by people who use drugs themselves to be most effective. As such, our study sought to investigate the harm reduction practices that work best for people who inject drugs from their own perspectives.

### Research objectives and questions

Our study had two major objectives: (1) to evaluate the Vidaview Life Story Board™ as a qualitative interview tool with PWID and (2) to explore the conceptualization of harm reduction and range of harm reduction practices among PWID in Ottawa. Results relating to the first objective are reported elsewhere. This article presents only data relating to the second objective. Several questions emerged from this objective: (1) Why do PWID use harm reduction strategies? (2) What types of strategies do PWID use to reduce harm in their daily lives? (3) What do PWID consider to be obstacles or facilitators to using harm reduction strategies?

This study explores the person-centred needs, values, and preferred outcomes of this marginalized community, and how to enhance equity in health and social services. By delineating a community-informed description of harm reduction, we expected to gain useful information to improve the efficiency of prevention and treatment programming for people who use drugs. Moreover, we anticipated that valuing community perspectives in the conceptualization of harm reduction would support agency among people who use drugs to collectively expand and shape their arsenal of harm reduction practices.

## Methods

### Participatory Research in Ottawa: Understanding Drugs

In 2004, Millson et al. [28] identified that HIV and hepatitis C rates among people who use drugs in Ottawa were among the highest in the country. With this in mind, a collaboration of researchers and community partners allied to gain a better understanding of the risk environment faced by people who use drugs in Ottawa through research which could inform policy and program development. To do this, the Participatory Research in Ottawa: Understanding Drugs (PROUD) Community Advisory Committee (CAC) was created in May 2012, and, while numbers of participants varied over time, included people with lived experiences^1^ using drugs (approximately 10), allies (approximately 4) and academic partners (approximately 4). One priority identified by the CAC was to highlight the social context and life experiences that surround harm reduction practices among PWID, and to outline a more culturally sensitive characterization of harm reduction, hence the PROUD team decided to conduct a sub-study to investigate this topic using the Life Story Board.

### Harm reduction through a structural approach

The primary focus of harm reduction interventions has been mainly centred on individual risk behaviour change [29]. Aligned with PROUD’s critical posture, we have chosen to consider the macro and micro contexts underlying the capacity of PWID to use harm reduction strategies, in accordance with Rhodes’ [29, 30] “risk environment” framework. Harm reduction interventions “are *social interventions*, subject to the relativity of risk and to variations in population behavior in different social, cultural, economic, legal, policy, and political environments. The relative success of individual, community and policy interventions are shaped by the risk environments in which they occur” [29]. Using this approach, we will consider individual actions as constrained by broader structural factors. Moreover, most harm reduction interventions have focused on managing behaviours that are likely to put *health* in peril. This research project aimed to move beyond health-focused strategies to include the subjective perceptions of PWID in regard to all outcomes that are important to them, as well as the impact of their environments.

### Research team

An innovative community-based participatory research methodology was used in which Peer Research Associates (PRAs)^2^ were directly involved in the study design, recruitment, qualitative data collection, validation of the research findings, and knowledge dissemination. This process followed a previously established model from the PROUD study [31]. PROUD Peer Research team members prioritized investigating harm reduction practices as an area of inquiry, while highlighting the importance of creating community-informed interview questions, and the need to adapt the Life Story Board for this context. The conceptualization and training process occurred within an 8-month span prior to beginning data collection.

Our research team was composed of researchers with lived experiences of current or former drug use, and academic researchers. Five CAC members took on the role of PRAs for this study. They were selected based on their lived experiences, interest in the study, and past research engagement. The PRAs were tasked with leading the interviews so as to create a culturally safe space and enable more open discussion of personal harm reduction practices used by the participants, including practices that may not be endorsed by service providers [5].

Another key member of the research team was the Peer Research Coordinator who acted as a liaison between PRAs and academic researchers, assisted with training and supporting PRAs, and provided cultural insight in data analysis and interpretation. All Peer Research team members were compensated monetarily for their involvement.

### Adaptation and training for the Vidaview Life Story Board tool

The Vidaview Life Story Board (LSB) tool was developed in order to help break down communication barriers in the therapeutic setting by facilitating discussion of difficult life experiences [32–35]. The LSB permits interviewers and participant to co-construct a visual “life-scape”, using a board, markers and customized picture-magnets, which depicts a participant’s lived experience, including personal, relational and temporal aspects.

In the present study, the tools’ creator provided several days of initial training to the research team on how to use the LSB interview tool. The research team adapted the tool by designing new magnets for the specific cultural context. All PRAs received training in qualitative interview methodology and research ethics, as well as extensive hands-on practice. For the objective of investigating community harm reduction strategies, the LSB tool was mainly used as an aid for information gathering and organization during the interviews, and the information displayed on the board largely echoed the information on the audio recording, thus we did not directly analyse the information on the board.

### Interview guide development

PRAs were involved in developing the semi-structured interview guide to explore the following topics: histories of personal harm reduction strategies, barriers and facilitators to implementing harm reduction strategies, and suggestions for enhancing harm reduction supports and services. The following description of harm reduction was used to start the interview: “Harm reduction includes all the ways you reduce your risk in your routines or decisions as an injection drug user”. This was stated to ensure participants were initially familiar with a generic description of harm reduction, and then they were encouraged to think about their own perspectives. In addition, during each interview with a participant, the PRAs provided examples of various perspectives by mentioning some of the personal practices they used in their daily lives to help manage their substance use. Initial questions included “When did you start injecting drugs?” and “Did you use any harm reduction practices when you first started injecting drugs?”, and the conversations continued to explore participants’ experiences until the present day. A separate semi-structured interview guide was created to explore participants’ experiences of participating in the study (e.g. use of the LSB and community-based participatory research methodology), and these results are reported in a separate manuscript.

### Sampling and recruitment

The Peer Research team members enrolled 24 participants in the study using street-based purposive sampling [31]. Because local people who use drugs are part of a relatively close-knit community and our Peer Research team had been involved in this community for many years, we anticipated it would be unusual for potential participants to be completely unknown to them. One or two of the Peer Research team members frequently knew the participants at the level of acquaintances. Inclusion criteria were as follows: participants needed to be at least 18 years of age, be living in Ottawa, and self-identify as having injected drugs in the previous 12 months. Participants also had to agree to disclose details about their injection drug use and harm reduction strategies. In addition, the recruiters specifically aimed for participants who had lengthy histories of injection drug use because they were expected to have more experience with harm reduction services and practices. The recruiters also purposely sought to include more women to ensure potential differences in perspectives among men and women were included overall. Compensation for time and travel was provided to each participant.

### Data collection

In July and August 2015, private rooms in three community health centres in central Ottawa were used to conduct the interviews. In the Ontario context, community health centres have a service delivery model that focuses on community development and typically includes both health care and social services to vulnerable populations [36]. These settings were chosen on recommendation of our Peer Research team because of their close proximity to the community of interest and because they provided spaces in which participants were likely to feel comfortable. In addition, due to the nature of the study and the potential for discussing difficult past events, it was arranged for a social service provider to be available on site to provide support to participants and PRAs if needed. This provider was not present in the room during any of the interviews.

Participants provided informed consent at the beginning of their interviews. Two PRAs conducted each interview: one PRA was responsible for asking questions while the other depicted participants’ experiences on the LSB. The board illustrated the evolution of participants’ drug use, social supports, and harm reduction strategies. In addition, a brief post-interview evaluation was conducted by the Peer Research Coordinator.

### Data analysis and interpretation

Each interview was audio-recorded, transcribed, and de-identified. Following transcription, the Peer Research Coordinator reviewed each transcript thoroughly to ensure content accuracy and for culturally relevant contextualization. As the participants’ vernacular contained language specific to the Ottawa drug user community, including information pertaining to certain generational or local contexts,^3^ aspects of the original interview content were non-interpretable to the academic research staff. Hence, this “cultural interpretation” process was critical for understanding the harm reduction information discussed throughout the interviews.

We used a conventional qualitative content analysis approach in that we inductively derived the codes from the data because there was limited literature detailing harm reduction practices from the perspectives of drug users themselves [37–39]. Questions and probes were open-ended and evolved according to the information participants shared. The analysis process initially involved immersing ourselves in the data to get overall impressions, followed by three academic research team members individually reading through a sample of transcripts to identify units of meaning, or codes. Triangulation involved the three coders’ discussing their perspectives at length in order to develop a comprehensive preliminary coding scheme and to identify any key differences. This coding list and the differences were then discussed and refined in collaboration with two Principal Investigators and the Peer Research Coordinator, who had also read the same transcripts. Using the identified list, one of the team members then coded every relevant statement, with labels for the codes emerging directly from the text, in the software program NVivo (version 10, 2012). After abstraction had been completed, the lead author developed a list of proposed sub-themes, including sample quotes to communicate the meanings associated with each sub-theme. Data interpretation was reviewed and refined by members of the team at multiple meetings, including attention to the grouping of sub-themes into different themes, and how to best label each theme. Finally, two focus groups were conducted with the PRAs to validate the prominent themes and sub-themes. We ensured credibility through the consistency criterion of reliability (i.e. we maintained a transparent decision-trail, with thought processes and interpretations discussed openly among academic and Peer Research team members) [40]. A risk environment framework was incorporated [29, 30] to help make sense of the ways these themes connect to the larger field of harm reduction. Research Ethics Board approval for the study was obtained from the Bruyère Research Institute and the Ottawa Health Sciences Network.

## Results

### Sociodemographic characteristics of sample

Twenty-four people who inject drugs were recruited and participated in the study. One participant had to be excluded due to an error in the audio recording process. Of the 23 participants included in analysis, the median length of the interviews was 82 min (interquartile range (IQR) = 46) including break times. Fourteen (61%) men and nine (39%) women participated, with a median age of 50 years (IQR = 7.5). The median length of time participants had been injecting was 29 years (IQR = 19.5). Participants had lived in Ottawa for a median of 30 years (IQR = 34). At the time of their interviews, 15^4^ (75%) participants lived in the Centretown, Downtown or Lowertown areas of the city, which are nearest to the majority of health and social services for people who use drugs. Furthermore, 13 (62%) participants lived in an apartment or house, and 14 (67%) considered their housing to be stable. A majority of participants reported using multiple types of drugs (e.g. opioids, crack/cocaine, benzodiazepines) in multiple forms (e.g. injecting, smoking, ingesting).

### Themes arising from the data

Several themes emerged from the data. Most notably, PWID described how their harm reduction strategies expanded beyond health and social service use; how their reasons for using harm reduction strategies were multi-dimensional; and the ways that structural factors inhibited or promoted their use of harm reduction strategies. While understanding how people with lived experiences conceptualize harm reduction strategies was the main objective, the other themes are informative in highlighting why such strategies are used and how micro and macro contexts influence their use. For each of the three main themes, only the most common or unique sub-themes are described in detail and highlighted with quotations, while other sub-themes are listed briefly.

### Harm reduction strategies expand beyond using health and social services

Participants described *how* they used specific strategies to incorporate harm reduction into their daily lives, and these strategies were grouped into two overarching themes: (1) accessing community health and social services and (2) employing personal practices.

#### Accessing community health and social services

Most participants mentioned that their harm reduction regimen included using services and supports in at least one of three community health centres located in the downtown core of Ottawa. The most common reasons for using these services included access to drop-in rooms, mobile vans, obtaining sterile equipment for substance use, moral support or positive social interaction with staff, information, a comfortable space or sense of connection with the community, and counselling. Although many of these reasons correspond to the primary intended purposes of the services, others are benefits incurred as a result of the health or social service delivery model. For instance, several participants made statements reflecting their appreciation for having a space in which they felt a sense of belonging to the community. As Jason^5^ explained:

I go there you know just to sit and have a coffee. I go to the back and I know everybody back there.

Likewise, participants often spoke about the importance of having moral support or positive social interaction with staff members at these centres. Kimberly described her experience in this way:

When I’m down sometimes I feel like using and stuff. I just come here and say ‘hi’ to whoever’s here. I just come and I feel better. […] Or I see this one and she makes me laugh. You know it doesn’t have to be talking about [using] or something.

On the whole, participants made it clear that frequenting community health centres had multiple purposes, including the convenience of having many health and social services offered in one location. Most importantly, however, it seemed that frequenting these centres often helped participants break social isolation.

In addition, all participants mentioned accessing services and supports at community-based health and social services other than those offered at the community health centres. In discussing their use of these services, it was common for participants to highlight particular individuals who had made a crucial difference in facilitating their access to such aid. For instance, Angela described the impact of housing support on her stability in the following way:

I have a little more stability going on, and if it wasn’t for [name of a service provider] putting me on the list for the [supportive] ‘housing thing’ when it first came out, I don’t know where I’d be today. I could probably still be out there on the street. Thank God for [her]!

Overall community-based health and social services are a crucial component of the harm reduction toolkit for PWID, especially when these services involve interactions with helpful and non-judgemental service providers.

Finally, opioid replacement therapy was a prominent harm reduction strategy that participants discussed at length in their interviews. Participants described opioid replacement therapy in both positive and negative ways, almost always in reference to methadone programs. Although methadone is clearly an important strategy for helping PWID to manage their daily lives, the mixed feelings about such programs appear to stem from several key issues, which we will address later in the results.

#### Employing personal practices

The second major theme of harm reduction strategies include personal practices that participants described using to manage their drug use and reduce the harmful impact it had in their daily lives. The most common practices included (1) using in moderation, including replacing one drug with another drug or adhering to prescription instructions, (2) engagement in the community, and (3) cognitive and behavioural strategies.

Employing moderation in using substances was described by participants as both a strategy used to reduce harm in their daily lives and as a lifestyle goal which they hoped to achieve. Participants talked about moderation through a multitude of terminology, including: “cutting down”, “keeping it down to a dull roar”, “dabbling”, “using very carefully with a lot less”, considering drug use to be “a treat”, “not using as much or as often”, or simply using “[j]ust very moderately”. Importantly, participants discussed the progress they had made toward improved control over their substance use and reduction of harms in their daily lives due to using tactics of moderation. As Patricia outlined:

[H]aving that three months behind me of moderation and trying to be aware of my decisions, it’s been easier since to keep things down a bit. Balanced.

Working toward increased moderation was the most common personal harm reduction practice that emerged from the data. Abstinence-based treatment models were not well appreciated by the participants, although a few participants considered such models to be helpful in some ways.

Participants also used moderation specifically in the way they tended to replace what they saw as more problematic substance use with the use of drugs that had less of an impact on their daily functioning. Steven described the importance of this practice for how he incorporated harm reduction into his life:

[I]n the last year what harm reduction means for me […] now it means trying to stay on softer drugs instead of harder drugs.

Marijuana was the most commonly mentioned drug participants used as a replacement for other substances that they felt caused them more problems. As Heather noted:

I can stay clean for a whole week. Like I mean I have no problems staying clean for a whole week. If he doesn’t get money from work, we’re good. We stay home, as long as we have that gram of weed. He comes home, he’s tired from work. We smoke a joint.

The next most common substitution drugs that participants mentioned were alcohol or cigarettes. Participants also noted the use of prescription medications for this purpose (whether obtained legally or illegally), including methadone, methylphenidate (Ritalin), hydromorphone (Dilaudid), aripiprazole (Abilify), venlafaxine (Wellbutrin), and Tylenol 3s. Furthermore, it is noteworthy that several participants indicated their use of replacement substances was with the intention to avoid using crack. Overall, participants’ replacement of their own most difficult-to-manage substances with other substances they felt they could regulate more easily was an important way they implemented moderation as a harm reduction strategy.

Participants also employed moderation through attempting to follow medication instructions as prescribed, despite enduring negative side effects. Participants noted efforts to minimize the use of other drugs that might interfere with their prescribed treatments. Heather mentioned that taking medication to manage her mental health symptoms was one of her key harm reduction practices:

[M]y harm reduction […] I’m bipolar so I’m on Abilify which is for depression, bipolar so […] that’s a fairly new one actually, so that kind of saves me every day. I don’t drink very much…

However, some participants also made changes to their medication intake to suit their everyday needs. For instance, sometimes participants ingested their prescription medications in non-specified ways (e.g. injecting or snorting), or they adjusted the procedures slightly (e.g. reducing or increasing dosages) in ways that they felt improved their ability for self-care. As Michael explained:

I take four of these a day. But I take one during the day and I take three at night. This works better for me. Because I find when I take two, it just makes me too dragged out. So when I take the three at night, I sleep like a baby…

PWID also described engagement in community activities as another essential harm reduction practice in their lives. Our participants mostly referred to being involved in community initiatives that served other people who use drugs or other marginalized populations in some manner, such as sharing their personal experiences relating to drug use, promoting services and identifying or requesting services that were needed, receiving overdose prevention training, or “needle hunting”,^6^ and these were also identified as ways to give back to the larger community. Some participants noted a desire to have more opportunities to participate in this type of work, in part, because helping others provided benefits to themselves.

Patricia described the powerful impact on her own well-being due to being able to help her peers:

I took the Naloxone training, the peer overdose prevention program […] and I’ve had […] successful resuscitations from respiratory arrest since then. That’s sort of given me a little bit of confidence and good feeling, to be able to help the people around me who are using. And to sort of, I guess remind myself of why I’m not wanting to do that anymore.

David went further to describe how his early involvement had led to increasing engagement due to being able to make a lasting change in the community:

It started, too, I guess the first time I started trying to do community stuff was when I was doing needle hunting […] And then I moved in this area, and I got involved with [another community organization]. But the needle hunting was neat. I remember the last year I was there, the government was thinking of closing it down. But we kept track of everything. And that summer I think we found something like 8000 needles […] So we had the proof and the numbers. Yeah. So then they kept it going.

The third crucial harm reduction practice that PWID identified involves the use of cognitive and behavioural strategies. With respect to cognitive strategies, almost all participants mentioned at least some instances of using increased awareness or self-reflection to help manage their substance use. Many of these mentions included the following types of thinking: explicitly trying to have more awareness, trying to make sense of things, reflecting on difficult or traumatic events (e.g. death, jail or prison, abuse), reflecting on drug use, addiction and harm reduction, and recognizing their progress over time. David noted that having more awareness was a helpful harm reduction practice:

Harm reduction. […] you’re trying to control the triggers and because addiction is so tricky, you’ll actually subconsciously do stuff and go some places […] I guess to me, it’s trying to be aware.

Similarly, Matthew outlined how reflecting on and learning to work through his emotions had contributed to gaining more control in his life:

I’ve come to a theory that I’m the only one who controls my emotions, not nobody and nothing around me can control how I feel. […] and if I allow myself to be depressed it means that I need to feel something so, I allow myself to feel it. […] it took awhile to get my brain to think like that.

Some participants also noted the benefits of maintaining a positive attitude or sense of humour in order to cope with harms arising from their drug use. Furthermore, participants who talked more about using cognitive strategies tended to be more hopeful about making progress in managing their drug use and lives overall.

Participants also considered certain behavioural practices among their harm reduction tools. They often described these practices in general terms, such as staying active or leaving the house, having a structure or routine, or simply keeping busy. Specific actions were also mentioned, including the following: athletics, working, traveling, volunteering, and participating in community activities. For Matthew, moving around meant challenging habits and thus reducing the possibility of developing harmful ones:

Well I just realized in the survey here that hitchhiking across the country was sort of a harm reduction; get away from one city, one type of drug, and then go to a different city to a different type of drug.

Several participants also mentioned that having a distraction of some sort helped them to better manage their drug use, and they suggested that community-based harm reduction services should incorporate more opportunities for people who use drugs to participate in activities or environments in which they are likely to be distracted.

Other harm reduction strategies that were mentioned included safe injecting/drug use practices (e.g. using sterile needles, disposal in biohazard containers), alternative drug use practices (e.g. re-using own needles only, disposal in garbage), not keeping cash readily available (e.g. direct payment of bills, giving cash away to others), and basic self-care (e.g. hygiene, sleep). On the whole, participants made it apparent that while making use of conventional harm reduction strategies, such as frequenting community-based health and social services, was an important component of their harm reduction arsenal, their personal harm reduction tactics were similarly critical to making progress in managing their drug use.

#### PWID have multi-dimensional reasons for using harm reduction strategies

Participants described multiple motivations for *why* they incorporated harm reduction strategies into their daily lives. One of the most common reasons cited was to improve their health. That is, many participants mentioned they were trying to better control their drug use because they wished to prevent or manage an illness such as an infectious disease, another physical health issue, an aging-related issue, or a mental health concern. As an example, Patricia noted her efforts to adjust her drug use habits in order to enhance the effectiveness of her treatment for hepatitis C:

So I’ve made an effort to not [inject drugs]. And if I’m using then it’s done in a different way. Part of that was driven by seeking treatment, medical treatment, for hepatitis last year.

In addition, for some participants, health was increasingly a reason to use harm reduction strategies (such as reducing the frequency of injecting) due to advancing age:

Peer Research Coordinator: Okay now, what’s more important to you now?

Caroline: It’s my health. […] I’ll always be a user if I don’t stop and what’s going to happen is, I’m [in my fifties] and I’m going to have a heart attack. I’m not stupid.

Aside from health-related motivations, another fundamental reason for using harm reduction strategies was to improve one’s social relationships. This mainly centred on attempts to re-kindle or maintain positive relations with one’s children, but also sometimes included references to relations with other family members, romantic partners, friends, and even pets. Steven discussed practicing harm reduction strategies because he was thinking more about his children:

I try and wear clean clothes, I try and eat three meals a day and I try in doing a little more self-care, and within the last week I haven’t used any hard drugs, and I think the guilt is coming in more for me maybe when I’m getting a little older now and that I am thinking more about my kids when I use hard drugs.

At the end of his interview, Steven explicitly linked his family to his use of harm reduction strategies:

And I think now, I think now I realize that my mom would be proud of myself that I have my addiction in check. And I’m sure my kids will come around, and the more I keep in the harm reduction, it’s a better chance I have of getting my kids back.

After moving into his daughter’s home, Michael also expressed the powerful impact of being given another chance by his loved ones:

I would not smoke in the house, like cigarettes, and I wouldn’t even bring it home. I wouldn’t even do it around the friggin’ house, you know? Like, she told me, she says: ‘The first time [Michael], I catch you or mom using, you’re gone and no questions asked’. So like, what’s more important, family, blood or a fuckin’ toke, right?

Some participants also noted that they were motivated to take care of themselves because of worries that their children might experience similar addiction-related challenges and they wanted to be available to support them.

A further key reason participants incorporated harm reduction strategies into their lives was because they were goal-oriented. Participants often projected themselves into the future by describing their projects or plans, and they indicated that reducing their drug use was connected to achieving other goals in their lives:

PRA: And I remember you saying that you work out, and you –

Donald: Yeah, I want to get back into that too. I was supposed to do back in Christmas. That was my goal. Quit smoking crack, and start working out and possibly go back to work. See if I could get a job.

Many participants also talked about how they were trying to return to normal or find balance. Jason described his progress in this regard:

But yeah and then now that 2015 came around it’s great, I’m doing volunteer, I’ve got a job, I cleaned right up. […] I ain’t smoking no more, my mom and dad big time, my family is back in my life, my son, everything is back to normal again.

Moreover, when participants discussed such improvements they often exhibited feelings of pride in their use of harm reduction strategies:

But that’s why I’m proud of myself […] well I mean, I have some sort of harm reduction. I’ve been clean for a week, I’m just [using] pot. (Steven)

Several participants mentioned additional reasons for using harm reduction strategies, including facing mortality (either of oneself or of another person), not wanting to do sex work, experiencing pregnancy, or avoiding contact with law enforcement. Overall, the majority of reasons participants discussed for employing harm reduction strategies in their lives centred on their relationships to others and themselves, which constitute part of their social environment [29].

### Structural facilitators and barriers for using harm reduction strategies

In order for the harm reduction strategies described above to be effective at reducing harm in the lives of PWID, many socio-structural aspects of their environments must be considered. First, our findings indicate that PWID face immense obstacles to implementing harm reduction strategies into their lives, including but not limited to rigid eligibility criteria or procedures, lack of accessible information or misinformation, societal discrimination and stigmatization, and negative affect. In addition, although the following list of barriers will not be discussed here because they have been described in other studies, we found that PWID face extensive issues with housing stability (an important part of the physical environment), financial stability (central to the economic environment), criminalization (largely a result of the policy environment), relationship problems and peer pressure (key to the social environment), as well as challenges related to physical or mental health issues [29].

A prominent barrier faced by the majority of participants was rigidity in eligibility criteria or procedures of many health or social services. The most commonly discussed area in which PWID felt they were expected to meet excessive criteria was in regard to the prescribing practices of physicians. As noted earlier, such inflexibility was most evident in opioid replacement programs. Other types of medications were also mentioned as being particularly restrictive to access, such as those for the purpose of treating mental illnesses, as well as those classified as painkillers or medical marijuana. Patricia mentioned that although she had maintained relative stability on pain medication for many years following a car accident, her prescription was taken away due to a blood test indicating that she had other drugs in her system:

[I]t was all pretty consistent until (a recent date). I got my meds pulled. Yeah, so after 14 years of being on [the medication]…

Likewise, Kimberly explained how rigid dosage procedures, in her case experienced when dosing during a prolonged hospital admission differed from those prescribed by her community physician, contributed to her relapse upon hospital discharge:

In the hospital I was getting […] Four and a half months, needles every four hours. […] You know so like I relapsed when I got out because he only put me back on 15 mg of methadone. I know maybe it sounds like an excuse, but […] while I was on methadone, I was successful for four years.

In regard to opioid replacement programs, for those participants who were on methadone maintenance therapy, problematic aspects of service design or delivery seemed to be a primary concern in their lives, regardless of whether they wanted to stay on the methadone or not. The issues participants experienced included difficulty receiving or maintaining carries^7^ (i.e. take-home dosages), restrictions in trying to change dosages, and the negative side effects (including more difficult withdrawal compared to other drugs). Some participants mentioned that they really wanted carries, yet their previous carries had been taken away too easily or they had never been given the chance to try one. This rigidity in permitting carries is detailed in Caroline’s discontented description of the program:

[T]he only reason I get up is to go and get that fucking methadone that I hate to go and do. Every day because I’m always dirty [drug test results show other drugs in system], you know? […] I’ve been on it for about 17 years and I’ve never had a fucking carry in my life. It’s like, just give it to me (laughter), I’m so sick of being on the program. […] Yeah right, like I really want to look at your face every day, to get a drink. Like there’s times when I don’t even go for 2 days, and not because I have anything to use, it’s because I don’t want to go.

Participants also often discussed the rigidity of methadone programs with respect to adjusting their dosages. Some participants mentioned being able to wean down to a lower dosage (when requested by their physicians or through their own self-regulation), whereas others mentioned that being given a lower dosage interfered with the stability they had achieved. Others indicated that their physicians tried to get them to change their dosage or to wean off of the methadone altogether despite their desire to maintain their current regimen. David described his experience, making it clear he wanted his needs and preferences to be taken into consideration:

[My methadone physician] actually wanted me to stop – work down to stop methadone. I’m going ‘No, I’m doing this shit until the day I die.’ Like, I’m not stopping. […] They don’t get it! […] And I’m going ‘Look it, if you’re gonna try to fucking cut me off like this, I’m gonna go somewhere else’. […]Well the thing is, too, I don’t get is, look, it’s fucking working, I’m not using. Why do you want to fucking cut me off now? It’s working! […] When I don’t have this, this is why I wanna use again. You know, right now it’s taking that craving away.

Furthermore, participants who were not on a methadone program described not wanting to take it or feeling lucky that they never had to, because either they had tried it and did not like it, had seen what it did to others, or even considered it a form of governmental social control. Other health or social services that were singled out for placing undue restrictions on drug users included mental health services (e.g. difficulty obtaining treatment unless abstinent), needle and syringe program practices (e.g. difficulty obtaining an adequate number of needles and syringes, or other equipment such as pipes), and accommodation services (e.g. difficulty following rules at shelters).

A further barrier that almost all participants mentioned was a perception of difficulty in obtaining harm reduction information in the community. Specifically, this mainly included instances in which participants indicated they were unaware of the existence of certain harm reduction services. This was evident in references to having a lack of awareness of services beyond the distribution of sterile equipment for substance use. A number of participants noted that while they initially started frequenting community health and social services for this purpose, it took longer than they felt it should have before they became aware of the additional services offered by these organizations (e.g. housing support, counselling). As Patricia described:

I became aware of the [mobile] van being available through media about needle exchange at a place downtown. Yeah, but it took a while to become familiar with some of the services.

Accordingly, some participants explicitly requested these organizations should make information about their services more accessible to the community:

Steven: Well I had support from them but I didn’t, I didn’t know about these programs.

PRA: But you wish that they had made it more, the information clearer.

Steven: More accessible.

The most prominent barriers not specific to health and social services were the pervasive anti-drug discrimination and stigmatization in society at large. The majority of participants mentioned experiencing discrimination or stigmatization because of their drug use, as well as for other reasons such as having an infectious disease, having a mental illness, being homeless, aging, being a woman, race/ethnicity, sex work, or having a criminal record. Such discrimination came from many different people they had contact with, including service providers, family or friends, and the general public. Angela’s experience depicts a poignant example of the effect of such widespread discrimination:

[T]hey were all saying ‘She’s nothing but a junkie’ […] you know ‘Look at her arms’. I remember the conversation, I could hear the conversation being said, like when I was in the hospital, like outside my room […] and it’s just […] I didn’t feel like I was worth much anyway.

Participants indicated that these types of experiences interfered with their use of harm reduction strategies by making them less likely to seek help from services, more likely to feel the need to lie to service providers, and more likely to hide their drug use from other people in their lives.

Finally, an important barrier emerged in regard to the influence of negative affect in participants’ lives. Despite attempts to incorporate harm reduction strategies into their daily lives, participants felt they were often treated with disrespect and condescension, including being judged or rejected.

Some of this negative affect occurred when participants had contact with service providers who they felt treated them in a condescending manner. Participants often indicated that they did not like it when service providers had no lived experiences with drug use or marginalization. They frequently described being disrespected, as if their opinions or feelings were not valuable. As Matthew noted:

I can’t deal with counselors because they’re friggin’ college educated with no experience and I hate how, them telling me how I’m supposed to feel. ‘Well, you should feel this.’ ‘Well, you know what? I don’t want to feel, and so please stop trying to make me feel that way.’

Other participants noted that negative affect stemmed from the judgement of their families:

PRA: Okay and are there people or circumstances that get in the way of you protecting or you practicing your harm reduction?

Steven: […] I had to throw the negative people out of my life. And part of those negative people was my family. My own family would bash me and throw me down so hard that I had to throw a lot of them out of my life…

In addition, participants reported a great deal of negative affect due to traumatic circumstances they had experienced (or were experiencing), including abuse and violence of various types or difficulty coping with the death of loved ones, which interfered with their use of harm reduction strategies. Note that a deeper exploration of these traumatic experiences was outside the scope of this study; hence, the methods were designed to retain focus on harm reduction experiences. As the interviews were research rather than therapy, and because of the potential to trigger trauma among our PRA team members, we purposely avoided exploring trauma during the interviews.

Overall, the magnitude and emphasis given to barriers in the interviews is a telling indication of the need to address the many socio-structural issues PWID face in order for ongoing harm reduction interventions to be as effective as possible.

Although facilitators were discussed much less often than barriers, there were still a few central aspects of participants’ lives which supported harm reduction strategies to thrive. In addition to finding strong evidence for several well-established facilitators that will not be detailed here, such as having support from loved ones (i.e. the social environment), stable housing (i.e. the physical environment), and steady income (i.e. the economic environment) [29], we also found that developing one’s self-esteem or self-efficacy, having continuity of care in health or social services, and having support from people with lived experiences, were all important facilitators for PWID.

One commonly mentioned facilitator was the development of one’s self-esteem or self-efficacy. Participants discussed many activities through which they increased their self-esteem or self-efficacy, including reconnecting with family, working or getting on the Ontario Disability Support Program (ODSP),^8^ gaining education or skills, improving their appearance, helping to make positive change in their community, obtaining recognition of the value of their lived experiences, and making progress in controlling drug use. For instance, Steven’s statement conveys the valuable impact of improving one’s physical appearance:

Steven: Yeah, I’m so, like as soon as I got my teeth, my confidence went out the roof!

Peer Research Coordinator: Yeah it’s funny how that happens.

Steven: And then I got off welfare, well I’m on ODSP now and I’m thinking ‘Okay, I’m on ODSP, I got my teeth, now I gotta get my glasses and I gotta get my motorcycle next year’.

For Michael, receiving positive feedback from his loved one was a powerful facilitator for using harm reduction strategies and making the most of the “last chance” he was being given, as expressed in the following conversation:

Michael: …I moved into my daughter’s house […] and […] my granddaughter, and it just gave me motivation to be just me.

PRA: It’s like a chance to be whatever…

Michael: Yeah, you know ‘last ticket to catch it’ sort of thing. I was thinking you know. And I was honoured by her asking me to give her away [at her wedding], that was awesome. With her own birth father, she says ‘[Michael], as long as I’m concerned you’re more of a father than my birth father’, and I said ‘Ah you shouldn’t be saying that’, you know what I mean. Wow!

Another facilitator for participants’ use of harm reduction strategies was to have continuity of care in health or social services. Participants often referred to the benefits of having consistent visits with a doctor or other service provider over time:

I got a counsellor once a week for an hour, and like I see my counsellor last week and I’m like ‘Hey, I’ve got a problem today’, and she goes ‘What?’, I’m like ‘I’ve gotta, I’ve gotta tell you about three hours of junk in one hour […] I’ll try my best but we only have an hour, let’s get going.’ (Everybody laughs) You know what I mean like, so it’s great that I have that every week because now I got somewhere to throw my shit right? (Steven)

The benefits of having continuity of services also sometimes involved frequenting one organization in preference to others. Furthermore, participants often discussed such continuity with reference to maintaining a positive relationship with a particular service provider:

Heather: I started getting introduced to Nurse [name of the nurse] in these years. […] and she’s been my God saviour. She was the only one that I can ever confide in and she’s the first one that I ever…

PRA 1: Nice.

Heather: Yeah she saved my life. She’s the one that got me diagnosed with bipolar.

PRA 2: Tell her that, it’s nice to hear that.

Heather: I do, I tell her all the time. I hug and kiss her all the time and she kisses me back. Oh yeah!

Finally, having support from people with lived experiences was another important facilitator. For example, there was evidence that secondary distribution of drug using equipment through peer networks was an important harm reduction practice. As Matthew described:

Yeah because you call the [mobile] van or the [other mobile] van out down where I live, because sometimes it takes a half hour for someone to get over there. So I make sure that I have enough that my friends can come knock on my door and ask for supplies.

Because waiting for sterile equipment can contribute to sharing drug using equipment and the subsequent risk of infection, secondary distribution as a back-up method may reduce such risk. In discussing the receipt of equipment directly from peers in his apartment building, Jason noted that the close proximity made this practice most convenient: “You didn’t even have to leave”.

In addition, the benefit of peer support was evident from participants’ reactions to being interviewed by their peers in the present study. As an example, Kimberly said the following in reference to the PRAs: “I’m grateful like you know, like for people like you guys…”

Accordingly, participants often indicated that they learned the most about harm reduction from their fellow peers:

Matthew: I think [my best friend] was the biggest harm reduction in my life because he introduced me to Ritalin. By the time I met him […] I’d let myself get a really bad cocaine addiction.

Participants also commonly discussed their desire for more avenues in which peer-to-peer support could occur among people who use drugs. Angela suggested this type of support would be a substantial improvement to the services available for this community:

[T]o have a little more people who are off the street now, maybe a little more community, like, meetings and stuff like that, not so much AA/NA but just meetings for us. […] ‘much’ (emphasized) more peer support, where we can sit down and talk about things that are going on in our lives and try and work it out as ‘a community’ (emphasized), like ourselves without inviting anybody from the outside in. […] Because those people don’t, like as far as I’m concerned, they don’t have a clue.

In sum, there are many facilitators that highlight not only the importance of reducing risks for PWID but also the need to foster growth of positive social relationships and self-progress over time. Taken together, the barriers and facilitators highlight the complex interplay of policy, social, economic, and physical risk environments that PWID face at both micro and macro levels [29].

## Discussion

Participants in our study chose to use harm reduction strategies for reasons that extended beyond the focus of mainstream supports and services on health management, including for the purposes of maintaining social relationships and working to pursue general life goals. Hence, they employed many alternative practices which they considered to be harm reduction in their daily lives. This finding corroborates limited previous research [5, 24, 25], yet the most prominent practices varied to some extent. The harm reduction strategies that were prioritized by our participants included using in moderation, becoming more engaged in the community, and exercising a range of cognitive strategies. Although both conventional and unconventional health-related strategies were also used, participants employed these personal practices more than other strategies. This may indicate the importance of harm reduction for PWID to address basic psychological needs of autonomy, competence, and relatedness [41], and why an expanded conceptualization of harm reduction should focus on the holistic well-being of both individuals and communities. An enhanced view of harm reduction^9^ should thus expand beyond the health-focused practices that are prevalent among community health and social services, to better acknowledge all practices which PWID believe to be helpful. Although some practices may not be currently endorsed by service providers (or included in public health framing), they may be an essential part of the harm reduction conceptualization by PWID. While we recognize that some practices may be difficult to evaluate from a strong evidence base, given our findings we further recommend that harm reduction policies and programs consider all practices that are meaningful to PWID, and at least acknowledge that they may be useful strategies for certain individuals despite lack of formal approval (or evidence) currently. Moreover, although we recognize the need to avoid promoting neoliberal “responsibilization” [42, 43]: advocating for more support of certain harm reduction practices that may improve drug users’ agency and self-care is important for fostering community solidarity [5, 21, 44]. Furthermore, this conceptualization of harm reduction is consistent with broader trends in the concept of health overall, “as the ability to adapt and self-manage” [45, 46].

Few other studies have asked people who use drugs to describe their day-to-day harm reduction practices in detail. Gowan et al. [5] outlined the ways their drug user group members considered the content of harm reduction to be subjective (i.e. involving working toward any positive goals), and that these principles influenced their use of daily self-care strategies. Although these authors did not provide an investigation of the types of strategies that people used, they noted that common strategies reported during their weekly sessions included “regular physical exercise, better nutrition, changing patterns of use to accompany daily schedules, paying rent on time, reconnecting with friends, and ‘drinking beer instead of the hard stuff’”. In one study that did conduct a thorough exploration of daily practices [24], participants’ self-care (but not explicitly harm reduction) strategies were categorized into five domains: “improve nutrition, increase physical activity, address medical concerns, regulate substance use, and reduce sexual risk”. Another study interviewed ethno-racially diverse gay and bisexual men and found five specific harm reduction strategies: “rationing, controlling or avoiding mixing, controlling quality, maintaining a healthy lifestyle, and following guidelines during substance use” [25]. As our study was focused on gathering community perspectives of harm reduction, in which we took explicit actions incorporating participatory methodology to promote information sharing and thinking “outside the box”, we identified a more comprehensive and community-informed list of strategies than in previous literature, which we hope will contribute to an enhanced understanding of harm reduction.

In addition to acknowledging the value of personal harm reduction practices utilized by PWID, it is imperative to address the structural environments in which all harm reduction strategies take place [29]. In particular, the major barriers included rigid eligibility criteria and procedures established by health and social services, lack of accessible information or misinformation concerning harm reduction services, societal discrimination and stigmatization, and pervasive negative affect. Our results confirm that although PWID are already performing many self-care actions in their daily lives, the risk environments they face tend to inhibit much of their efforts. For instance, the manner in which participants discussed taking methadone illustrates the major issues associated with their policy environment. This strategy was presented as a critical component of many participants’ overall harm reduction regimens, yet the “rigid constraints” imposed on these programs render them limited and in need of reform [11, 47], a finding that supports the importance of considering theoretically effective interventions from the view of people’s lived experiences [48]. The issues seem to arise from care procedures that lack personalization or negate the involvement of people who use drugs in decision-making; this indicates the need to incorporate a person-centred care model into opioid replacement therapy programs. Although revising health care standards and guidelines is a necessary step to do this, limits of the programs may also stem from the social environments within which service delivery occurs, thus provider practices may need to be targeted for reform as well. Such changes would be consistent with Health Canada’s Best Practices for Methadone Maintenance Treatment [49]—that programs should adopt a non-punitive and therapeutic approach, including in regard to urine toxicology screening for the use of other drugs. In Ontario, however, some of the issues may stem from strict regulations or practices around urine drug screening and take-home doses, outlined in the College of Physicians and Surgeons of Ontario’s Methadone Maintenance Treatment Program Standards and Clinical Guidelines, in accordance with drug laws and physician monitoring practices [47]. Thus, a key conclusion from this study is the need to address such inhibitory factors at the same time as promoting self-care among PWID, as the latter may be futile without the former.

Our results also suggest that in order to create and sustain supportive environments it would be useful to endorse certain facilitators, such as the development of self-esteem or self-efficacy, continuity of care in health or social services, and increasing (or offering) peer support. Enhancing these facilitators in the lives of PWID could reduce some of the barriers they encounter, promoting environments in which harm reduction practices would thrive. For example, as our participants suggested, PWID would benefit from having access to more peer-led services, in particular because this allows them to obtain social support and information within an anti-stigmatizing space that is safe from judgement and discrimination. As such, we recommend that current services and supports work to integrate people with lived experiences of drug use in increasing capacities, such as by implementing consumer participation initiatives [50]. Considering the barriers and facilitators together with the array of harm reduction strategies and reasons for using them, there is a need to highlight the importance of both personal and collective actions as part of the efforts enhancing harm reduction’s influential role in present society.

There were several limitations to our study. First, structuring the interviews around timelines of people’s injection drug use led to some difficulties in disentangling temporal references throughout the transcripts. Although the PRAs tried to maintain a chronological sequence, this did not always work well as participants sometimes had difficulty following the timeline. They would jump back and forth across time which resulted in confusion for both the PRAs during the interviews and the academic researchers who coded the transcripts. These difficulties were likely due to many factors, including participants’ personal style or cognitive states, aspects of participants’ histories, or fatigue with the longer interview process. Future research could consider a different approach to focusing the questions, such as using a life events or topic-based structure, or perhaps tracking a shorter span of time or having the questions pertain only to the present. Another limitation was the fact that certain questions were not always asked or answered in a clear enough way that would allow researchers to interpret with confidence what was said. Despite having the Peer Research Coordinator review all transcripts and de-code culturally specific information, there were still instances in which the audio lacked sufficient context to enable understanding. Sometimes this occurred because the PRAs and participants were acquainted with each other prior to the interviews; hence, they were familiar with aspects of each other’s lives, which meant some discussions included minimal spoken explanation. Moreover, additional information seemed to be lost from the transcripts due to the heavy use of non-verbal behaviour (e.g. gesturing, facial expressions) during the interviews. Finally, given the characteristics of our participants, our sample included more women and had an older age compared to other studies of PWID, and accordingly had a lengthier history of drug use and more housing stability. Thus, it is possible they also had more experience (and success) with using harm reduction strategies.

## Conclusions

PWID have multi-dimensional reasons for using harm reduction strategies, which include a focus on general well-being rather than only on managing health or reducing risk. Furthermore, PWID employ many personal practices in their daily lives that extend beyond using health and social services, and these actions highlight the importance of agency and community building in a community-informed conceptualization of harm reduction. However, such harm reduction practices employed by PWID are not fully effective due to multiple socio-structural obstacles. Thus, improving the effectiveness of harm reduction practices cannot rely solely on improving self-care strategies among people who use drugs; rather, collective community action is required to address the many barriers they endure and to sustain supportive environments for harm reduction to thrive. Our findings demonstrate the need to increase equity of health and social services for drug users, including focused efforts on reducing anti-drug stigmatization, criminalization, and promoting harm reduction principles and actions.

Furthermore, our study demonstrates that the perspectives of people who use drugs are critical to conceptualizations of harm reduction because they highlight what this community wants or needs, as well as what seems to be working or not working, in a culturally sensitive manner. Our results underline that “one size does not fit all” when it comes to harm reduction strategies. Hence, harm reduction services should better reflect the multiple preferred goals and outcomes of the affected community, as well as de-medicalizing services so that they will be more relevant to the everyday lives of people who use drugs. Community health and social organizations should incorporate these results to improve engagement and retention of people who use drugs in services, especially through the implementation of support from people with lived experiences.

## Abbreviations

CAC: Community Advisory Committee
HIV: Human immunodeficiency virus
LSB: Life Story Board
PRA: Peer Research Associate
PROUD: Participatory Research in Ottawa: Understanding Drugs
PWID: People who inject drugs

## Footnotes

1 To reflect the diversity of lived experiences among people who use drugs, our community team members would prefer that the term “lived experience” be pluralized when referring to multiple individuals.

2 We use the title “Peer Research Associate” in this article to designate an individual who has both lived experience and training in research activities. Although our community team members would prefer to remove the word “peer” from this title, we agreed that it is beneficial to use in order to connect with similar literature.

3 The “generational” context refers to information specific to different time periods (e.g. 1980s or 1990s). The “local” context refers to information specific to certain people or places which may only be known to people who use drugs. Sometimes local information was combined with generational information (e.g. a location in Ottawa where people used drugs in the late 1980s), making interpretation difficult and requiring the services of a person with lived experience.

4 Note that because not all participants answered every question, percentages were calculated based on the number of participants who responded to each question.

5 To protect participants’ confidentiality, all names used in this article are pseudonyms.

6 Needle hunting refers to searching the city (especially areas considered “hot spots” for drug activity) to safely collect used needles, syringes, or other drug paraphernalia that was discarded on public property rather than properly disposed of in a biohazard container. Many community organizations have teams who complete this activity, and community members will volunteer or are paid for participating in the clean-up process.

7 A “carry” refers to permission for individuals on Methadone Maintenance Therapy to take a limited supply (up to six doses per week) of methadone home to be ingested at their subsequent regular dosage times. In Ontario, individuals must submit to supervised urine screening once or twice a week (at fixed or random intervals) and the results must indicate no illicit drug use for at least 2 months before they will be given a carry. A physician also needs to deem that the individual has not shown behaviours or symptoms of “problematic drug use” for at least 1 week prior to being given a carry. Individuals who do not have a carry are required to go to their pharmacy for daily observation to consume the dose. For more information, see https://www.cpso.on.ca/uploadedFiles/members/MMT-Guidelines.pdf.

8 The Ontario Disability Support Program includes stable income support for people who meet the definition of a person with a disability: “you have a substantial mental or physical impairment that is continuous or recurrent, and is expected to last one year or more and, your impairment directly results in a substantial restriction in your ability to work, care for yourself, or take part in community life and, your impairment, its duration and restrictions have been verified by an approved health care professional” (http://www.mcss.gov.on.ca/en/mcss/programs/social/odsp/income_support/IS_Eligibility.aspx). People consider this form of support preferable to Employment Insurance (also referred to as welfare) which provides only temporary income support under more restrictive conditions.

9 Although we use the term “harm reduction” to describe all the strategies people who inject drugs use in their daily lives to reduce the potential for harm, we acknowledge that not all community members or scholars appreciate this term. Moreover, it may not be the best term to encompass all of the practices participants described in our study. One concern is that the term may imply that achieving a *neutral state* is a desired outcome, whereas our participants clearly indicated that their goals include working toward positive outcomes (i.e. not only reducing negative outcomes). This conceptualization might be better described as “building strength”, or more simply as “self-care”.
